# Behavioral Medicine Methods in Treatment of Somatic Conditions

**DOI:** 10.1155/2020/5076516

**Published:** 2020-11-03

**Authors:** Joanna Białkowska, Jakub Juranek, Joanna Wojtkiewicz

**Affiliations:** ^1^Department of Psychology, Sociology of Health and Public Health. School of Public Health, Collegium Medicum, University of Warmia and Mazury, Clinical University Hospital in Olsztyn, 10-900 Olsztyn, Poland; ^2^Institute of Psychology, Polish Academy of Sciences, Jaracza 1, 00-378 Warsaw, Poland; ^3^Department of Pathophysiology, School of Medicine, Collegium Medicum, University of Warmia and Mazury, 10-900 Olsztyn, Poland

## Abstract

**Background:**

The aim of this article is to present a short review of noninvasive, nonpharmacological treatment methods used in somatic illnesses that fall under the umbrella of approach called behavioral medicine.

**Methods:**

The narrative review method was applied in the study. Science paper databases, including PubMed, had been used to retrieve papers on therapeutic methods used in clinical setting that meet the broad criteria of behavioral medicine definition as stated in the Charter of International Behavioral Medicine Society

**Results:**

Main groups of methods, disorders in which they are being employed and their effectiveness, have been identified.

**Conclusions:**

Behavioral medicine is grouping treatment methods and interventions that hold large potential for clinical setting. Two groups of methods can be distinguished by the scrutiny and level of evidence gathered in their effectiveness assessment; for biofeedback, guided imagery, and hypnosis techniques, comprehensive evidence reports in the framework of U.S. Evidence Synthesis Program exist. Meditation techniques, disclosure therapies, and relaxation methods are less well assessed. Broader employment of behavioral medicine therapies in clinical setting is possible after addressing two major problems in the field, which are deficiencies in quality evidence of effectiveness for many of the methods and their insufficiencies in underlying therapeutic mechanism knowledge.

## 1. Introduction

Since before the emergence of modern medicine, it has been observed that behavior and states of mind have relation with health and illness. This important understanding, broadly present in ideas of ancient thinkers like Aristotle, Galen, and Islamic Golden Age Persian physicians like Abu Zayd Al-Balkhi and `Ali b. al-`Abbas al-Majusi (latinized: Haly Abbas), had been somewhat subsided over time with the successful raise of mechanistic paradigm in medicine that followed works of academics such as William Harvey and his explanation of heart functioning as a pump or Rene Descartes notion of mind and body as utterly separate “machineries” [1, 2]. As late as the first half of 20^th^ century, the reemergence of awareness that crucial links exist between behavior, mental processes, and body functioning was witnessed. With rise of behaviorism and works of figures such as Ivan Pavlov, previously undescribed connections, like between central nervous and digestive system, mediated by conditioning, became exposed. Accumulation of such knowledge leads to eventual advent of psychosomatic medicine [3] and later distinct from its forebearer—behavioral medicine [4, 5].

Behavioral medicine is interdisciplinary field, integrating and using psychosocial, behavioral, and biomedical knowledge relevant to health and illness, in an effort to understand fundamental biobehavioral mechanisms of well-being and serve the needs of clinical diagnosis, intervention, disease prevention, and health promotion [6–8]. It incorporates conceptual meanings of the term “behavioral” on four distinctive levels, as elucidated by Weiss and Schwartz [5, 9]. At the primary level, “behavioral” means relation with concepts and techniques derived from behavioral therapy, as the behavioral medicine field was first inspired by advances in operant conditioning and the emergence of biofeedback theory. The second level pertains broader health relevant concepts and techniques, which are subsets of wider, more general psychological knowledge, like ideas originating from positive psychology and findings coming from affiliated discipline of health psychology. The third level is “behavior” conceptualized also in wider context of social sciences like sociology or anthropology, enabling appreciation of variables like social support or spirituality in context of health. The fourth level translates “behavioral” as functional, meaning interest in common, high-level principles that apply to the functioning of different systems at various levels of reality, keeping an open door for potentially usable and significant wisdom coming from far related, or unrelated, fields, spanning from physics to ecology.

The interests and interdisciplinary character of behavioral medicine is responsible for its strong interrelation with other health oriented disciplines and subdisciplines that have rooting in psychology, namely, psychoneuroimmunology [10], psychoendocrinology [11], psychooncology [12], and the broadest, earlier mentioned, psychology of health [13]. A common trait of all these health-related fields is emphasis on holistic *bio-psycho-social* (or—as extended by recent propositions—*bio-psycho-socio-cultural* [13]) approach to health [14].

Due to its interdisciplinary aspect, the essence of combining knowledge from broad spectrum of areas with the aim to facilitate synergy, blending skills and ideas, behavioral medicine has proved to be currently, and ventures to be in the future, a fertile ground for deriving useful and innovative strategies for the prevention, diagnosis, and treatment of a wide range of health problems. Much potential is being recognized in this field in terms of new treatments and improving cost-effectiveness of health care systems [15].

Realization of behavioral medicine potential depends on proper research and availability of information regarding existing solutions in that framework. The aim of this paper is to review contemporarily applied and researched interventions used in the treatment of somatic conditions, falling into the domain of behavioral medicine by its definition. Although formal birth of this discipline had taken place just in the year 1977 (with the founding Yale Conference on Behavioral Medicine [16]), treatments included in this review often have longer history. The inclusion criterion in this paper was given treatment matching the scope of behavioral medicine definition, as outlined in the Charter of International Behavioral Medicine Society [7], and not its history or theoretical origin.

## 2. Review of Therapeutic Method Groups

Interventions falling into behavioral medicine category are primarily nonpharmacological techniques, originating from the appreciation of the interrelations between the body and mind. Many of them are based on the employment of principles of learning (conditioning), or recognition of the importance stress (of various origins) has on organism (including its significant impact on functioning of the immune system). Treatment approaches include a group of biofeedback methods, relaxation techniques (also biofeedback supported relaxation), disclosure interventions, hypnosis, guided imagery and visualization techniques, meditation, and bulk, stress-management programs. Some of the methods amalgamate and share common features, benefiting from nonidiosyncratic mechanisms; for example, types of meditation can include focus on briefing, visualizations, and relaxation, and several of the methods possess common aspect of being founded on principles of learning. Many of the separate techniques are often combined and administered under a joint umbrella of stress-management programs [17–24].

Two separate groups of methods can be distinguished by the scrutiny and level of evidence gathered in their effectiveness assessment; for biofeedback, guided imagery, and hypnosis techniques, comprehensive evidence reports in the framework of U.S. Evidence Synthesis Program exist, and so unified style summarizing tables depicting condition and outcome area effectiveness are provided. The remaining methods' effectiveness and area of application are reported in the text or different form of tables.

## 3. Biofeedback

Associated with the founding roots of behavioral medicine movement in science and connected with the first sense of term “behavioral”—meaning related to behavioral therapy and methods—biofeedback techniques rely primarily on the principles of operant conditioning. The essence of the wide group of these interventions is learning by individual how to voluntarily control body processes—which are commonly falsely considered involuntary—for health benefits or symptom control. That is why, the term “treatment,” emphasizing the curing goal of these methods, but accentuating passivity, is better replaced here with the word “training,” highlighting the need of active patient participation in the learning and thus curing process. Biofeedback methods substantially employ equipment that converts physiological input from the body into auditory or visual signals that constitute the feedback for the patient. With such feedback, a person can clearly learn how their mental states are resembled in the physiological state of the system targeted in particular biofeedback intervention and try modifying and managing it, typically with support of a qualified practitioner. Instrumentation used in different biofeedback methods measures muscle activity, skin temperature, electrodermal activity (sweat gland activity), respiration, heart rate variability, blood pressure, brain electrical activity and blood flow [25]. Biofeedback techniques are employed in two modes: alone (less often) or combined with stress management, relaxation, psychotherapeutic and other techniques [17].

One of the most common biofeedback trainings involves electromyography (EMG), conveying feedback regarding muscle activation, the intensity of muscle contraction, and manifestation of muscle fatigue [26]. Electromyography feedback in clinical setting has been pioneered in the 1920s by Edmond Jacobson [27–29]. It has been tried since, to treat various conditions, among which are arthritis [30], asthma [31], Bell's palsy [32], cerebral palsy [33, 34], hand dystonia [35, 36], repetitive strain injury [37], and spasmodic torticollis [38]. It has proved to be the most efficacious in the treatment of various kinds of chronic pain [25], headaches (tension, migraine, or of mixed origin) [39, 40], temporomandibular disorder [41], and constipation [42].

Another biofeedback method is thermal biofeedback. It is associated with self-regulation of skin temperature [43]. This type of biofeedback is usually performed with the use of skin temperature sensors, called thermistors, attached to fingers, with visual or tonal feedback to the subjects. Thermal biofeedback is usually administered with other modalities of biofeedback techniques and demonstrated to be useful in the treatment of Raynaud's disease [43], headaches [44], and chronic pain conditions [25, 45].

The idea behind yet another biofeedback mode—skin conductance feedback—is to deliver person information about sweat gland activity, which is closely correlated with sympathetic nervous system functioning [46]. With very short response time falling in the range of below two seconds, skin conductance activity (SCA) feedback—known also as EDA (electrodermal activity) or GSR (galvanic skin response) feedback—provides superb sensitivity to emotional changes [25]. This biofeedback technique receives a lot of attention as a complementary epilepsy treatment with encouraging outcomes reported regarding its efficiency [47–50]. Other medical uses have been and are being studied, including employment of this technique in support of controlling glucose levels in type 2 diabetes [51], treatment of Tourette syndrome [52], and headaches [53].

Another example of biofeedback technique is heartrate variability (HRV) feedback. It involves monitoring either heart rate alone or heart rate plus respiration. Data regarding heart rate is collected via electrocardiograph (EKG) monitors or plethysmographic sensors on the finger or earlobe [25]. It is has been tried and used in a variety of disorders, with multiple potential pathways for its therapeutic effects proposed [54, 55]. Best results were reported in treating recurrent abdominal pain [56], chronic muscle pain [57], and cyclic vomiting [58].

Arguably, the most popular biofeedback application could be electroencephalogram (EEG) neurofeedback (EEG-NFB)—a method which takes advantage of the fact that particular brain wave patterns correlate with specific disorders and syndromes [59]. Improvement in functioning in these disorders may be possible due to the changes in the amplitudes or overall proportion of brain wave types. These changes occur as a result of teaching the patient to strengthen the desired brain wave frequencies, amplitudes, or potentials and inhibit the undesirable ones. There are three main protocols used in EEG-NFB interventions. The most commonly used is the frequency training, in which the goal is changing the power ratio of the EEG frequency bands. It is mostly used in attention deficit hyperactivity disorder (ADHD) and autism spectrum disorders treatment [60, 61]. Slow cortical potential (SCP) is a second kind of popular neurofeedback protocol, based on modulating specific (slow) event-related potentials (negative or positive); it is also mainly used in ADHD. In somatic disorders, a third protocol is however most widely used. It is coherence training. The aim of this method is promoting the coherence of activity (frequency as well as amplitude and phase) of different brain regions. Theoretical foundation for this protocol lay in the fundamental observation that specific decoherence (or distorted connectivity) often accompanies particular disorders.

EEG neurofeedback is widely used to treat epilepsy [62]; it is also being administered in novel capacities to treat conditions such as fibromyalgia [63] or chronic prostatitis [64] and is commonly adopted in rehabilitation, for example, in patients with amyotrophic lateral sclerosis (ALS) [65], stroke [66], spinal cord [67], and traumatic brain injuries [68, 69].

Doubts about utility of neurofeedback methods exist and stem mainly from three reasons, as comprehensively outlined by Thibault and Raz [70]. One reason is the deficiency of methodologically high-level, large, quality-controlled, and randomized studies proving its effectiveness in vast amount of conditions it is applied to. Another cause is existence of the so-called sham neurofeedback effects: placebo-like, positive results, obtained using feedback taken from the activity of other than the trained person brain or similarly irrelevant source [71]. The third reason is lack of widely acknowledged and established mechanisms that would explain the claimed therapeutic benefits. The research work addressing these problems is however already being conducted, with good outlook regarding accumulation of better evidence considering effectiveness and establishment of the psychological and neural mechanisms underlying clinical benefits [72].

Current EEG neurofeedback techniques serve as an inspiration for developing more advanced brain-subject feedback loops, employing the functional Magnetic Resonance Imaging (fMRI) systems. This relatively new (emerging in year 2003) biofeedback expanse area is still mostly field of ongoing research with potential yet to be evaluated, less a ground of mature clinical applications [73–75]. The surge of interest in this subject has been reported in the last decade, and promising new technical advances and protocols are in development. Examples of these are the decoded neurofeedback (DecNef) technique—aiming at inducing a specific activity pattern within a target brain region—and functional connectivity-based neurofeedback (FCNef), in which the goal is changing functional connectivity between different regions [74].

In the beginning of the 21^st^ century, the Task Force of the Association for Applied Psychophysiology and Biofeedback plus the Society for Neuronal Regulation formulated criteria for designated levels of evidence-based clinical efficacy of psychophysiological interventions. They span from level 1—not empirically supported—to level 5—efficacious and specific [76]. According to Yucha and Montgomery [25], who rated evidence of efficacy in conformance with the abovementioned criteria in 2008, there were eleven (see Table 1) conditions for which biofeedback training could be considered either as “efficacious and specific” or as “efficacious” (level 5 and level 4) and further nine conditions in which it was rated as being probably efficacious (these include arthritis, diabetes mellitus, and urinary incontinence in males), with additional eighteen were possibly efficacious (level 2). The one condition which fits into the “efficacious and specific” level is urinary incontinence in females. General effectiveness (level 4) has been stated for anxiety, attention deficit hyperactivity disorder (ADHD), chronic pain, constipation in adults, epilepsy, headache in adults, hypertension, motion sickness, Raynaud's disease, and temporomandibular disorder (TMD).

More recent report compiled under the auspices of the US Veterans Health Administration Evidence Synthesis Program (EVP) [77, 78] concluded (see Table 2) there is strong, high-confidence evidence for biofeedback intervention efficacy in clinical conditions like pain resulting from migraine or tension headaches and urinary incontinence in men after a prostatectomy. Moderate evidence was found supporting beneficial effects in fecal incontinence and stroke treatment. Contrary to summary by Yucha and Montgomery [25], hypertension, woman urinary incontinence outcomes for fibromyalgia, was described as providing no benefits, but with low-confidence evidence base. For the vast majority of other biofeedback clinical applications, the authors of the report abstained from conclusions stating insufficient data.

## 4. Hypnosis

Hypnosis is a method of inducing in an individual a state of hypnotic trance, a mode of deep relaxation and focus with increased suggestibility and suspension of critical faculties. In this special state of consciousness, two types of suggestions can be made to the affected person: direct hypnotic suggestion, made while the person is in trance, which alters behavior or perception while the trance persists, and posthypnotic suggestion that alters behavior or perception after the trance ends. Typical hypnotherapy consists of hourly or half-hourly sessions with a practitioner; additionally, group hypnosis interventions also exist. Some medical health specialists introduce hypnosis in 10-15-minute sessions along with their regular clinical work. In some cases, patients are taught to induce self-hypnosis (which is also accomplished by posthypnotic suggestion) [78].

Similarly to other techniques described here, hypnosis has been tried in many medical contexts. Effectiveness has been reported in irritable bowel syndrome [79], headache treatment [80, 81], chronic pain, and anxiety [82]. There is probable effectiveness of hypnotherapy as relief support in oncological and odontological procedures and promising outlook in interventions in diabetes and the human papilloma virus [83]. There are reports suggesting a positive role hypnosis can play for the functioning of the immune system [20]. The report developed in Evidence Synthesis Program supported conclusion about evident positive effect of hypnotherapy in cancer-related care and obesity treatment. Probable positive effects were pointed in irritable bowel syndrome and reducing anxiety related to undergoing medical procedures [77] (see Table 3).

Some authors suggest hypnosis is underused in medical setting, considering its already known utility [84]. Application and effectiveness of hypnosis in medical setting depend on obeying fundamental rules, which encompass the need and willingness of the patient to get rid of the complaint, believe in the method, and trust in the practitioner applying it [85]. An important factor worth mentioning in the context of hypnotic treatment effectiveness is specific individual difference characteristic: susceptibility to hypnotic suggestion, referred to as hypnotic suggestibility (also known as hypnotizability or hypnotic susceptibility [86]). It is a trait relatively stable, measurable by means of several standard procedures, partially heritable [87]. Some authors suggested it to be a core variable in responsiveness to hypnotherapy in clinical setting [88]. Meta-analysis approach, however, exhibited only small and moderate effects of this trait in the mediation of hypnotic therapy effectiveness [86]. Research shows most medical patients regardless of hypnotic susceptibility may benefit from the integration of hypnotic therapies into their medical care, as so hypnotizability testing is rather recommended to detect cases of low susceptibility, who may benefit more from alternative approaches [87].

## 5. Guided Imagery and Visualisations

A precise definition of guided imagery has been stated by Rider and Achterberg [89] in their 1989 paper, where they describe it as “the internal experience of a perceptual event in the absence of the actual external stimuli.” Guided imagery is thus invoking one or more senses experience with the use of imagination and guidance of specially designed instruction. Concentrating attention on envisioned images and scenarios reduces uncomfortable thoughts and feelings and distracts the mind from unwanted and negative state [90].

There are reports suggesting stress reduction effects and elevation of the immune system functioning in people treated with the guided imagery technique [23]. Additionally, pain relief effects had been observed [91]. In 2005, Roffe and colleagues [92] in their studies researching guided imagery effectiveness in cancer patients concluded it as playing an adjuvant, psychosupportive, and comfort increasing role, but with no compelling evidence suggesting positive effects on physical symptoms.

The EVP report [93] notes a high level of confidence for usefulness of guided imagery techniques in arthritis/rheumatic disease and potential positive effects in interventions regarding Parkinson's disease, stroke, cancer, and menstrual disorders (see Table 4). A beneficial role has been also noted in supporting patients treated in Intensive Care Units (ICU) and persons undergoing cardiac surgery procedures.

## 6. Relaxation Techniques

Relaxation techniques are a large set of methods that are commonly being divided into two main categories: somatic- (also called “physical”) and cognitive- (also called “nonphysical”) based approaches. The physical category includes for example pure behavioral relaxation training, progressive muscle relaxation, deep breathing, the Alexander technique and Feldenkrais method (education techniques aiming at establishing a heightened awareness of movements), and Mitchell's simple physiological relaxation [94–96]. Cognitive or nonphysical approaches include, among others, autogenic training—first described already in 1932 [97]—and Benson's method [98, 99] which are more focused on accomplishing the relaxation state by a person following the specially crafted instructions, rather than physical work with the body.

Relaxation techniques have been administered to support curing and treatment of various clinical conditions (see Table 5). Multiple relaxation methods are used as an adjuvant to treat pain. Progressive muscle relaxation (PMR) (combined with guided imagery) in cancer patients brought positive results in pain relief [100]. The Mitchell method—administered for one month—managed to reduce symptoms accompanying fibromyalgia [101]. An older review of usefulness of relaxation techniques in acute pain management showed however poor performance of these methods [102]. Relaxation techniques proved to be useful in treatments of nausea of different origins, chemotherapy-caused [103, 104] or pregnancy-related [105]. Beneficiary effects have been reported in Chronic Obstructive Pulmonary Disease (COPD) (regarding both respiratory functions improvement and better psychological wellbeing) [106] and in ischaemic heart disease recovery efforts [107]. Relaxation techniques are commonly used as accompanying methods to alleviate symptoms and improve psychological health in illnesses, like edema, anxiety, and depression in postmastectomy lymphedema patients [108] or distress in people with diabetes [109].

## 7. Disclosure Interventions

The theoretical basis of the disclosure intervention methods lays in the supposition that inhibition of psychologically significant thoughts and feelings has adverse health consequences, [19, 110]. In the 1980s, Pennebaker et al. in their correlational studies uncovered a pattern suggesting that not disclosing stressful experiences to others (e.g., spouse death, Holocaust survivorship, incest, and personal failures) is related to poorer health [111, 112]. Pennebaker and Beall aimed later at reversing emotional suppression by developing an experimental paradigm in which individuals could disclose and process stressful experiences [113, 114].

The most commonly employed technique in this approach is “written emotional disclosure” or “expressive writing” that consists of 15 to 30-minute long sessions (often repeated in consecutive days) of writing essays on personally experienced stressful events. Sometimes instead of written, verbal descriptions are used [115], or the disclosure takes place by means of drawing or combination of writing and drawing [116].

The disclosure-based methods have been employed in many health problem contexts, exhibiting modest benefits in chronic pain (limited in rheumatoid arthritis, far better performance in fibromyalgia, mixed in headaches, cancer pain, pelvic pain, and abdominal pain) [113] and noticeable positive effects in wound healing [117, 118]. Earlier, grander reviews in the form of meta-analysis produced mixed conclusions regarding disclosure interventions efficacy. Frisina and colleagues [119] reported significant positive improvements in health in clinical populations, with greater impact on physical than psychological health outcomes. In contrast, Meads and Nouwen concluded the nonexistence of emotional disclosure effects and suggested the reassessment of the method validity [120]. What appears to be of crucial importance in the context of disclosure effectiveness is the consideration of additional moderating factors such as emotional expressivity and baseline affect [121, 122], cognitive-emotional processing [123], social constraints [124], and ethnicity and form of the writing instruction [125].

## 8. Meditation and Mindfulness Interventions

Meditation is a practice in most general terms grounded in controlling focus of attention. It is referred in the classical languages of Buddhism—Sanskrit and Pali—as *samādhi*, etymologically meaning “gathering the mind and placing it upon an object” [126]. It can be categorized on the broadest level into three types. Concentration meditation depends on focusing attention on an object until quieting of the thoughts occurs in a subject's mind. Mindfulness meditation on the other hand emphasizes nonjudgmental openness to contents that arise in the mind spontaneously, not following and suspending reactions to them, promoting expanded awareness of present moment. Contemplative meditation is a mixture integrating both approaches, trying to combine focus with mind openness [127, 128].

Mindfulness meditation is the basis of mindfulness interventions. These interventions share the core premise of mindfulness—a process of openly attending, with awareness, to one's present moment experience—however referred by name to intervention schemes and programs [129].

A substantial body of research evidence, comprehensively reviewed by Sampaio and colleagues, cites important effects of meditation on not only body function parameters like reduction in respiratory frequency, slower heart rate, and reduced electrical conductivity of the skin [130] but also significant neuroendocrine and neurochemistry effects including structural changes to brain areas [131]. Other worth noticing positive influences on health and treatment that had been observed encompass a damping effect on T CD4+ lymphocyte reduction in adults infected with HIV-1, increased NK (Natural Killer) cytolytic activity [132–134], reduced risk for mortality in patients with coronary disease [135], and numerous evidence for enhancement in mood and well-being, decrease in stress, anxiety, and depression symptoms in healthy and ill individuals [134, 136, 137].

## 9. Discussion

A short review of the most prominent groups of therapeutic methods in the field of behavioral medicine shows a large potential in the context of contemporary health care system use. One of the main advantages of the reviewed methods is their intrinsic noninvasive and pharmacology free essence. This characteristic positions them as promising alternatives for treating conditions in which pharmacological or other types of therapies are burdened with harmful risks. A good example here might be the problem of pain treatment. Currently, opioid analgesics are associated with one of the biggest US public health crises: the opioid crisis. Research showed that overwhelming majority of the opioid abusers began their addiction with prescription medications, primarily for chronic pain [138, 139]. Pain, and particularly chronic pain, is the condition for which many of the reviewed behavioral medicine treatment methods proved to be efficacious, with a high level of confidence. Various biofeedback techniques, especially EMG, HRV, and thermal feedback, had been successfully administered in pain treatment—including chronic pain. Utility in this context has also been reported for relaxation techniques, with some more moderate evidence also found for hypnosis and guided imagery. Broader implementation of the best techniques from behavioral medicine repertoire at specific pain problems in clinical populations can bring relief to patients reducing the need of using medication, thus lessening the risks and potential of negative consequences associated with painkiller drugs. It is worth mentioning that usage of alternative, nonpharmacological treatments of pain was much more common in years before the emergence of opioid crisis [140].

Another distinctive advantage of behavioral medicine treatments is the fact that many of them are founded on learning processes and rely on training. This provides possibilities unavailable to other therapies, like surgery or pharmacotherapy. Persons treated with the learning-based therapies acquire skills and knowledge, meaning that in very many cases (those not requiring specialized apparatus like fMRI feedback), they can apply the treatment to themselves whenever necessary on their own. Furthermore, they keep the trained skill and can use them long after they acquired it and also possibly apply it outside the original illness context. Relaxation, expanding mindfulness, and self-hypnosis are skills which can beneficially be applied in health and even outside of health related context, for the rest of life. This means contact with some of the behavioral medicine techniques means arming people with useful techniques they can apply for promoting their health and improve wellbeing in the long term. This in turn might equal more years lived in better health and less strain on health care system.

Another leverage associated with many of the reviewed methods is their big potential in the context of convenient, remote, offsite use (eHealth) [141, 142]. Used case examples have already been reported for mindfulness, relaxation techniques, and hypnosis administered via tools such as smartphone applications or tele-sessions in which the therapist uses video call capabilities to remotely conduct intervention with a patient [129, 141, 143]. Automation and saving potential are clearly visible here.

Behavioral medicine-derived medical treatment field is also affected with problems. The issue repeatedly mentioned in the literature is deficiency of high-quality research that would enable drawing definitive conclusions regarding the effectiveness of many of the considered methods [71, 93]. Till now, only minority out of plentitude of mentioned here interventions had been conclusively confirmed or rejected as useful therapies in given conditions. Until effectiveness will be established with high-quality evidence, the dissemination of behavioral medicine therapies in medical setting will understandably not be high. Another major problem, along insufficiency of effectiveness evidence in many of the use cases, is similar deficit of empirically sound explanations of mechanisms underlying therapeutic benefits. Solving these two major deficiencies is a key prerequisite for enhancing the use of behavioral medicine treatment approaches in clinical setting.

## 10. Conclusions

A behavioral medicine framework provides wealth of methods for use in the treatment of multitude somatic health conditions. Those alternative approaches hold substantial potential in terms of reducing risks and unwanted negative consequences in cases where they can effectively substitute pharmacological or invasive therapies. Another useful area of employment for these methods is complementary and supporting role along the main treatment. Broader dissemination and popularization of these techniques can occur when insufficiencies regarding evidence of effectiveness and underlying curing mechanisms will be tackled.

## Figures and Tables

**Table 1:** Medical conditions for which evidence suggests biofeedback therapies are “efficacious and specific” (level 5) and “efficacious” (level 4) according to a work by Yucha and Montgomery [25]. Types of biofeedback therapy approaches are listed next to each condition.

Efficacy evidence level 5—efficacious and specific	Condition	Method
	Urinary incontinence in females	Pelvic floor muscles biofeedback
Efficacy evidence level 4—efficacious		
	Anxiety	Biofeedback (various modalities, including EMG and EEG)
	Attention deficit hyperactivity disorder	EEG biofeedback
	Chronic pain	Biofeedback (EMG, thermal & EEG biofeedback—utilization for given disorder best determined by consulting literature for that specific condition)
	Constipation in adults	EMG and manometry biofeedback
	Epilepsy	EEG biofeedback
	Headache in adults	EMG biofeedback
	Hypertension	Thermal, electrodermal response (EDR), heart rate, EMG, or direct blood pressure biofeedback
	Motion sickness	Galvanic skin response (GSR) biofeedback
	Raynaud's disease	Thermal biofeedback
	Temporomandibular disorder	EMG biofeedback

**Table 2:** Health conditions for which biofeedback interventions had evidence of a positive effect on diagnosis-related, secondary, or global outcomes according to the U.S. Department of Veterans Affairs Evidence Synthesis Program (ESP) report published in 2019 [77].

Condition	Diagnosis-related outcomes	Secondary outcomes	Global outcomes
Fecal incontinence	X		
Headache	X	X	X
Stroke	X		
Urinary incontinence after prostatectomy	X		X

**Table 3:** Health conditions for which hypnosis interventions had evidence of a positive effect on diagnosis-related, secondary, or global outcomes according to the U.S. Department of Veterans Affairs Evidence Synthesis Program (ESP) report published in 2019 [77].

Condition	Diagnosis-related outcomes	Secondary outcomes	Global outcomes
Anxiety in cancer patients	X		
Breast cancer care	X		
Obesity/weight loss	X		

**Table 4:** Health conditions for which guided imagery interventions had evidence of a positive effect on diagnosis-related, secondary, or global outcomes according to the U.S. Department of Veterans Affairs Evidence Synthesis Program (ESP) report published in 2019 [77].

Condition	Diagnosis-related outcomes	Secondary outcomes	Global outcomes
Arthritis/rheumatic diseases	X		
Cancer		X	

**Table 5:** Health conditions treated with relaxation techniques with reported positive effect outcomes [101–107].

Condition	Method
Cancer pain	Progressive muscle relaxation
Fibromyalgia	Mitchell method
Nausea—chemotherapy-caused	Progressive muscle relaxation
Nausea—pregnancy-related	Benson's method
Chronic Obstructive Pulmonary Disease	Various relaxation techniques
Ischaemic heart disease recovery	Various relaxation techniques
